# Management of Acute Diarrhoea in Primary Care in Bahrain: Self-reported Practices of Doctors

**Published:** 2007-06

**Authors:** Abdulrahman Y. Ismaeel, Khalid A.J. Al Khaja, Awatif H.H. Damanhori, Reginald P. Sequeira, Giuseppe A. Botta

**Affiliations:** 1 Department of Microbiology, Immunology and Infectious Diseases; 2 Department of Pharmacology and Therapeutics, Arabian Gulf University, Manama, Kingdom of Bahrain; 3 Ministry of Health, Manama, Kingdom of Bahrain

**Keywords:** Compliance, Cross-sectional studies, Diarrhoea, Acute, Drug therapy, Oral rehydration solutions, Oral rehydration therapy, Bahrain

## Abstract

This nationwide study was conducted to assess the extent of adherence of primary-care physicians to the World Health Organization (WHO)-recommended guidelines on the use of oral rehydration therapy (ORT), antimicrobials, and prescribing of other drugs used in treating symptoms of acute diarrhoea in Bahrain. A questionnaire-based, cross-sectional survey was carried out in primary-care health centres. During a six-week survey period (15 August–30 September 2003), 328 (25.2%) completed questionnaires were returned from 17 of 20 health centres. In a sample of 300 patients, oral rehydration salts (ORS) solution was prescribed to 89.3% (n=268) patients; 12.3% received ORS alone, whereas 77% received ORS in combination with symptomatic drugs. Antimicrobials were prescribed to 2% of the patients. In 11.4% of the cases, rehydration fluids and other drugs were given parenterally. The mean number of drugs was 2.2+0.87 per prescription. In approximately one-third of the patients, three or more drugs were used. Primary-care physicians almost always adhered to the WHO guidelines with respect to ORT and antimicrobials. However, in several instances, ORT was prescribed along with polypharmacy, including irrational use of drugs for symptomatic relief. Effective health policies are needed to reduce the unnecessary burden on the healthcare system.

## INTRODUCTION

Acute diarrhoeal diseases are an important public-health problem worldwide. Diarrhoea continues to be a major cause of hospitalization and death of young children and has major economic consequences (1,2). In the United States alone, an estimated 211-375 million episodes of acute diarrhoea occur each year, resulting in more than 900,000 hospitalizations and 6,000 deaths (3). In developing countries, the rate of diarrhoeal illness is 2-3 times higher than that in developed countries (4), and most studies on acute diarrhoea carried out during the last decade were limited to children aged less than five years. These studies have mainly addressed the perceptions and practice of oral rehydration salts (ORS) solution therapy, prevalence of diarrhoea, social determinants of diarrhoea, and microbiological considerations of causative pathogens. Since the national guidelines for the management of acute diarrhoea have not yet been established in Bahrain, the treatment of diarrhoea is largely based on the World Health Organization (WHO)-recommended guidelines (2). Acute gastroenteritis is considered one of the common complaints in the primary-care setting in Bahrain; nonetheless, its management has never been evaluated. This cross-sectional study evaluated the extent of adherence of primary-care physicians in Bahrain to the WHO-recommended guidelines in terms of the use of ORS solution therapy, use of antimicrobials, and prescribing of drugs for treating symptoms of acute diarrhoea.

## MATERIALS AND METHODS

### Setting

The Kingdom of Bahrain, with a population of 650,604, is a group of islands located in the Arabian Gulf. The Directorate of Health Centres, which has a network of 20 well-structured primary-care health centres spread across the country, provides free curative and preventive care services to both citizens and expatriates, including dispensing of essential drugs. In addition to medical services delivered at the morning session, 10 of these health centres provide daily evening and night services for 3-8 hours. On a trial basis, one of these health centres currently provides health services on a 24-hour basis. The number of primary-care physicians in each of these health centres varies from four to eleven. Patients requiring special investigations and consultations with specialists or admission are referred to the Salmaniya Medical Complex which provides secondary-tertiary care. The Salmaniya Medical Complex also serves as a teaching and research centre for health professionals. Based on drug-prescribing patterns, a cross-sectional survey of self-reported practices of doctors in treating acute episodes of diarrhoea was carried out in the 20 primary-care health centres in Bahrain.

### Source of data and study population

A questionnaire was used for collecting data during 15 August–30 September 2003. All patients, attending the primary-care health centres, with acute diarrhoea during this period were included as the sample for the study. Acute diarrhoea was defined as a passage of loose or watery stool, usually at least three times in a 24-hour period (2).

Questionnaire: A descriptive questionnaire was developed and pre-tested by primary-care physicians. The questionnaire consisted of the following components: (a) information about the physicians; (b) demographic characteristics of patients (age, gender, and nationality); (c) time elapsed between onset of diarrhoea and attending health centres in days; (d) frequency of passing stool per day; (e) consistency and nature of stool; (f) complaints of patients; (g) history of medications; (h) physical examinations of patients; (i) microbiological investigations; (j) management of diarrhoea with pharmacological (i.e. ORS solution therapy and/or other therapeutic drugs) and non-pharmacological measures (i.e. assurance, diet advice, education, increased intake of fluid, stopping medication in the case of iatrogenic diarrhoea, sick leave/rest).

The questionnaire was distributed to primary-care health centres through the office of Chief of Medical Services for Primary Health Care, Directorate of Health Centres/Primary Health Care, Ministry of Health. The primary-care physicians were requested to complete the questionnaire while examining patients complaining of acute diarrhoea.

This descriptive study used the WHO guidelines for evaluating the extent of adherence to treatment: the adherence was broadly categorized into (a) adherence to history-taking and physical examinations, (b) adherence to rehydration principles, and (c) adherence to prescribing.

Data were analyzed using the SPSS software (SPSS/Pct, version 10.0). Descriptive statistics, such as percentage, mean, and standard deviations were used for describing the study variables.

## RESULTS

During the six-week period of the study, 328 (25.2%) completed questionnaires were returned. The number of questionnaires returned from 17 of the 20 health centres ranged from 3 to 47 (mean+SD=17.4+14.6) per health centre. Seventy (37.8%) of 185 primary-care physicians participated in treating 328 episodes of diarrhoea. The number of patients with diarrhoeal episodes seen per physician was 4.7+3.9 (median 4; range 1-20). Of the 328 patients with acute diarrhoeal episodes, 58.2% were male, and 90.9% were Bahrainis.

The mean age of the patients with acute diarrhoea was 24.8+15.3 years with a median age of 24 years (range 0.1-65 years). The distribution of the patients in the study population according to age was as follows: children <12 (22.4%), adolescents 13-19 (13.8%), adults 20-44 (51.5%), middle-aged 45-59 (10.1%), and elderly >60 (2.1%) years. The incidence of acute diarrhoea was most common in adult patients.

The characteristics of diarrhoea, findings of physical examinations, and laboratory investigations requested are shown in Table 1. The mean time elapsed between onset of diarrhoea and attending health centres was 2.2+1.9 days, and the mean frequency of passing stool per day was 5.0+2.6. Two hundred thirty-nine (72.9%) of the 328 patients with acute diarrhoea complained of watery stools, and eight (2.4%) complained of bloody diarrhoea, whereas stool characteristics were not specified in 81 (24.7%) patients. Based on the signs and symptoms of dehydration, the physical examinations revealed that only 48 (14.6%) patients had mild-to-moderate dehydration, whereas 270 (82.3%) had no signs of dehydration.

**Table 1:** Characteristics of diarrhoea, clinical and laboratory findings

Diarrhoea-related parameters	Number	Percentage
Characteristics of stool		
Watery	239	72.9
Blood mixed	8	2.4
Not specified	81	24.7
Physical examinations		
Normal (non-dehydrated)	270	82.3
Mild dehydration	45	13.7
Moderate-severe dehydration	3	0.9
Not specified	10	3.0
Laboratory investigations		
Routine stool examination	36	11.0
Stool culture	3	0.9
Routine stool examination and culture	10	3.0
None of the above	230	70.1
Not specified	47	14.9
Complaints		
Abdominal pain	146	44.5
Vomiting	33	10.1
Fever	10	3.0
Abdominal pain + vomiting	61	18.6
Abdominal pain + fever	27	8.2
Vomiting + fever	12	3.7
Abdominal pain + vomiting + fever	18	5.5
None of the above	12	3.7
Not specified	9	2.7
Time (days) elapsed between onset of diarrhoea and attending health centres (mean+SD)		2.2+1.9 (326)
Frequency of passing stool per day (mean+SD)		5.0+2.6 (320)

Figures in parentheses indicate the number of patients

Of the 328 acute episodes of diarrhoea, 300 (91.5%) patients were on pharmacological intervention, and six (1.8%) were on non-pharmacological interventions (assurance, education, dietary advice, fluid intake). In 22 (6.7%) cases, patients’ management was not specified.

The prescribing patterns for the treatment of 300 episodes of diarrhoea are shown in Table 2. In a sample of 300 patients, ORS was prescribed to 89.3% (n=268); 12.3% received ORS alone, whereas 77% received ORS in combination with symptomatic drugs and/or intravenous fluids. The mean number of drugs per prescription was 2.2+0.9, whereas the mean number of drugs per prescription containing three or more drugs was 3.3+0.6 (Table 3). The categories of drugs prescribed for the treatment of acute diarrhoeal episodes are presented (Table 4). Rehydration fluids and other drugs were given parenterally in 11.4% of the cases.

**Table 2:** Prescribing patterns for treatment of 300 diarrhoeal episodes∗

Prescribing pattern	Number of drugs					Total	Percentage
	1	2	3	4	5		
ORS only	37	-	-	-	-	37	12.3
IV only	6	-	-	-	-	6	2.0
Drugst only	17	4	1	-	-	22	7.3
ORS + IV	-	1	-	-	-	1	0.3
Drugs + ORS	-	141	66	13	-	220	73.3
Drugs + IV	-	2	2	-	-	4	1.3
Drugs + ORS + IV	-	-	3	5	2	10	3.3
Total number	60	148	72	18	2	300	99.8

∗Of 328 patients, 6 (1.8%) were on non-pharmacological interventions, and in 22 (6.7%), patients' management was not specified; tlnclude tablets, suspensions/syrups/drops, pills, capsules, and injections IV=Intravenous fluid; ORS=Oral rehydration solution

**Table 3:** Number of drugs prescribed per episode of diarrhoea among 300 patients∗

Prescription characteristics (Number of drugsf)	Diarrhoeal episodes	Percentage
1	58	19.3
2	149	49.7
3	72	24.0
4	18	6.0
5	2	0.7
6	1	0.3
Total	300	100
Number of drugs/prescriptions (mean+SD)		2.2+0.9
Number of >3 drugs/prescriptions (mean+SD)		3.3 + 0.6

∗Of 328 patients, 6 (1.8%) were on non-pharmacological interventions, and in 22 (6.7%), patients' management was not specified; f Include tablets, suspensions/syrups/drops, capsules, injections, oral rehydration solution, and intravenous fluids

**Table 4:** Drugs prescribed for treatment of 300 diarrhoeal episodes∗

Type of drug	Number	Percentage
Fluid and electrolyte therapy		
Oral rehydration solution	268	89.3
Intravenous fluid		
0.9 % sodium chloride	8	2.7
0.18% sodium chloride + 4.3% dextrose	3	1.0
Antimicrobials		
Amoxycillin	3	1.0
Cephalexin	1	0.3
Metronidazole	2	0.7
Symptomatic drugs		
Antidiarrhoeals	21	7.0
Antispasmodics	242	80.7
Antipyretic/analgesic	36	12.0
Antiemetics	36	12.0
Prokinetic	13	4.3
H_2_-receptor antagonist	7	2.0
Antacids	5	1.7
Antiflatulents	2	0.7
Intramuscular injection		
Antispasmodics	15	5.0
Antiemetics	8	2.7

∗Of 328 patients, 6 (1.8%) were on non-pharmacological interventions, and in 22 (6.7%), patients' management was not specified Antidiarrhoeal=Loperamide; Antispasmodics=Fixed dose combination of chlordiazepoxide + clidinium bromide (Librax¯), hyoscine butyl bromide (Buscopan¯), fixed dose of methylscopolamine + buta-barbital drops (Restropinal¯ drops); Antipyretic/analgesic=Paracetamol; Antiemetics=Promethazine (Phenergan¯), prochlorperazine (stemetil¯); Prokinetic=Metoclopramide; H_2_-receptor antagonist= Ranitidine; Antiflatulents=Carminative mixture

## DISCUSSION

The main goal in the management of acute diarrhoea is to prevent dehydration (if there are no signs of dehydration), treat dehydration (when it is present), and prevent nutritional insufficiency, particularly in children, by feeding during and after diarrhoea (2). The first two objectives can be achieved with ORS solution therapy which is accepted as the gold standard to achieve clinically-efficacious and cost-effective management of acute gastroenteritis (2,5). Unless the patient is comatose or severely dehydrated, ORS solution is recommended regardless of the causative agent and age of the patient (2,3) because ORS solution therapy is less expensive, often just as effective, and more practical than intravenous fluid (4). Our study revealed that 89.3% of the patients received ORS solution (12.3% received ORS alone, and 77% received ORS in combination with symptomatic drugs). The prescription rate of ORS solution in our study was comparable with (6), and considerably higher (7,8–11) than the rates reported by previous primary care-based studies from other countries. This finding suggests that, in Bahrain, the primary-care physicians adhered to the WHO guidelines in the management of acute diarrhoea, particularly with regard to the use of ORS solution therapy. However, such practice was found in several instances to be marginalized by prescribing irrational drugs used for symptomatic relief in acute diarrhoea.

Routine prescribing of antimicrobials to patients with acute diarrhoea should be discouraged, because: (a) the majority of cases of acute diarrhoea are due to viral and non-invasive bacterial infections (2,4,5); (b) the duration of mild, self-limited diarrhoeal illness is not decreased by use of antimicrobials (2,4,5); (c) the irrational use of antimicrobials results in wasting of resources and risks adverse reactions (2) and can increase production of Shiga toxin by enterohaemorrhagic Escherichia coli O157:H7 (5) and cytolethal distending toxin by Campylobacter jejuni (12); and (d) it can lead to increased antimicrobial resistance, particularly when the sensitivity and resistance patterns of the potential underlying causative microorganism is not determined (2,5,13). We observed that antimicrobials were used conservatively at a rate of 2% (Table 4), which was significantly lower than the rates reported elsewhere (7,8–11), and conformed to the guideline recommendations (2,3,5).

The use of antidiarrhoeals is contraindicated in children due to concerns about toxicities (2,3,5,14). Withdrawal of loperamide syrup formulation from Bahrain Drug Formulary by the mid-1990s reflects the awareness of health policy decision-makers. Although antidiarrhoeals, such as loperamide, have a limited role for symptomatic relief in adults with acute diarrhoea (2), we observed that 7% of the patients with diarrhoea received loperamide as an adjunct therapy (Table 4). However, loperamide should be considered only in adults who are not febrile or having bloody/mucoid diarrhoea.

Prescription of prokinetic metoclopramide to patients with acute diarrhoea is a controversial issue. The use of prokinetics in 4.3% of the patients, with a potential to cause diarrhoea (15), cannot be justified (Table 4). We believe that metoclopramide is irrationally used, in particular, if it was indicated for its antiemetic effects in patients with gastroenteritis, since diarrhoea is one of the potential adverse effects of metoclopramide (16).

Antiemetics, such as promethazine (Phenergan¯) and prochlorperazine (Stemetil¯), are contraindicated, particularly in children with acute diarrhoea (2,5). This is because: (a) correction of dehydration per se stops vomiting and restores appetite (2); (b) sedation induced by these antiemetics may interfere with ORS solution therapy (2); and (c) a post-marketing surveillance has revealed that promethazine can produce potential fatal respiratory depression in children aged less than two years (17). We observed that antiemetics were prescribed to 36 (12%) patients with acute diarrhoea (Table 4). Four (19%) of 21 patients who were on promethazine therapy were children aged less than two years. The use of promethazine in infants has been restricted due to safety concerns (18). Moreover, four (26.7%) of 15 patients who were receiving prochlorperazine were adults who received prochlorperazine intramuscularly. A high incidence of dystonias has been reported with intramuscular prochlorperazine (19,20).

Antimuscarinic-antispasmodics as symptomatic drugs were prescribed to 80.7% of drug-treated episodes of diarrhoea (Table 4). Despite their effectiveness, they are no longer considered appropriate for use in infantile colic (21). Restropinal¯ drops, a fixed-dose combination of methylscopolamine (antimuscarinic antispasmodic) and butabarbital was given to 35.3% (6/17) of the infants and to 40% (24/60) of the children aged 1-12 year(s), in our survey. Sedation induced by butabarbital—a component of Restropinal¯—should be considered since it may interfere with the ingestion of ORS solution, as is the case with the sedative promethazine (Phenergan¯).

The relatively short-time interval between onset of acute diarrhoea and visiting health centres (2.2+1.9 days) and lack of dehydration in the vast majority of (n=270, 82.3%) patients in our study are important considerations (Table 1). Plausible explanations for these findings may be related to: (a) a community awareness about diarrhoea and its hazards; (b) a well-organized network of primary-care health services; and (c) health-seeking behaviour of the population.

Characteristics of diarrhoea, especially low frequency of passing stool per day and low proportion of patients with dehydration or bloody diarrhoea (Table 1), suggest that the vast majority of cases of acute diarrhoea were mild and self-limited. This explains why requests for microbiological investigations and other laboratory tests were judicious, and the physicians complied in general with the published guidelines (3–5).

Polypharmacy practice is well-known to be associated with drug-related adverse reactions, medication errors, clinically significant drug interactions, and an increased risk for admission of patients (22). Based on the drug-prescribing patterns, we observed that polypharmacy, defined as prescriptions containing three or more drugs, comprised one-third of the cases, with a mean value of 3.3+0.6 drugs per prescription (Table 3). This polypharmacy practice seems to be related to free medical services, including dispensing essential drugs (23).

A few caveats on the study design and interpretation of the results are to be considered. To what extent the observed findings of the study are generalizable and reflect a nationwide practice is uncertain. Also, how the adherence to the treatment guidelines influenced the outcomes, such as treatment-failure rate, mortality, and adverse events from medications, need to be explored. This study was a survey of self-reported practices of doctors in the management of diarrhoea. Their actual practices may not always be the same as reported in the questionnaire. The rigorous WHO guidelines for the treatment of diarrhoea may not always be appropriate due to the complexity of some diarrhoea cases. The appropriateness of antimicrobials prescribing cannot be assessed without stool culture results.

However, based on the findings of the study, it can be concluded that primary-care physicians well adhered to the WHO guidelines with respect to the use of ORT and antimicrobials. In several instances, although ORT was used, polypharmacy and prescribing of irrational symptomatic drugs were also observed. The findings suggest that effective health policies are needed to reduce the unnecessary burden on the healthcare system.
